# Is it coincidental or correlative between reversible splenial lesion syndrome and atrial septal defect? A case report

**DOI:** 10.1097/MD.0000000000022920

**Published:** 2020-10-23

**Authors:** Jiangang Li, Yingcong Chen, Jianxue Liu, Xingsheng Mai, Shaohua Jing

**Affiliations:** a Departments of Neurology; b Departments of Ultrasonic of Medicine; c Departments of Imaging, Baoji Municipal Central Hospital, Baoji, Shaanxi, China.

**Keywords:** reversible splenial lesion syndrome, the splenium of corpus callosum, atrial septal defect, right-to-left shunts

## Abstract

**Rationale:**

Reversible splenial lesion syndrome (RESLES) is a recently identified clinico-radiological syndrome, the etiology is miscellaneous. Atrial septal defect (ASD) as an underlying etiology for RESLES has not been reported. We first report a rare case of RESLES associated with ASD. The clinical, radiological, and ultrasonic profiles were presented and the pathophysiological mechanism was analyzed.

**Patient concerns:**

A 23-year-old man presented with headache, drowsiness, occasional paraphasia, and paroxysmal dry cough. Brain magnetic resonance imaging (MRI) on admission showed an ovoid isolated lesion in the splenium of corpus callosum, which exhibited hyperintensity on diffusion-weighted imaging and hypointensity on apparent diffusion coefficient, and completely disappeared on the follow-up MRI 14 days later. ASD was found by transthoracic echocardiography, Right-to-left shunts were detected on color Doppler of transesophageal echocardiography, and microemboli were captured by transcranial Doppler ultrasound.

**Diagnoses:**

According to his clinical history and imaging results, we confirmed the diagnosis of RESLES associated with ASD.

**Interventions:**

The patient was treated by oral aspirin and lopidogrel sulfate to inhibit platelet aggregation. In addition, oral nimodipine to suppress vasoconstriction.

**Outcomes:**

After 14 days treatment, all the symptoms presenting on admission resolved completely. Subsequently, a repair surgery of ASD under thoracoscopy was successfully performed.

**Lessons:**

To our knowledge, this is the first reported case of ASD may be an underlying etiology for RESLES and need require an etiotropic treatment.

## 1 Introduction

Garcia-Monco et al. first used the term ‘reversible splenial lesion syndrome (RESLES)’ to define a typical radiological characteristic and excellent prognosis syndrome in 2011.^[1]^ The pathophysiology of RESLES is still unclear, and the etiology is unknown. Besides infection, epilepsy, drug withdrawal, metabolic disorders, high-altitude cerebral edema, and other familiar pathogenesis,^[1]^ some rare pathogeneses such as migraine,^[2]^ cerebral venous thrombosis,^[3]^ and anti-VGKC autoantibody^[4]^ have been gradually reported. To the best of our knowledge, this is the first case to report atrial septal defect (ASD) as an underlying etiology for RESLES.

## 2 Consent

The present case report was approved by the Ethics Committee of Baoji Municipal Central Hospital (Baoji, China) and the patient provided written informed consent for publication of the case and accompanying images.

## 3 Case report

**Figure 1:**
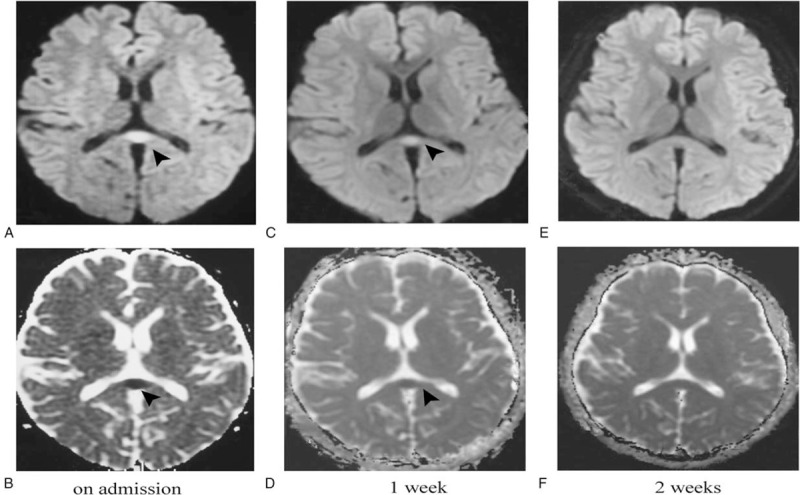
Brain magnetic resonance imaging (MRI) changes of the lesion in the splenium of corpus callosum (SCC) among the initial images and at 1-week and 2-weeks of follow-up. Initial MRI images show an ovoid isolated lesion in the SCC (arrow), exhibiting hyperintensity on diffusion-weighted imaging (DWI) (A) and hypointensity on apparent diffusion coefficient (ADC) (B). The lesion signal (arrow) weakened on a review MRI a week later (C, D), and completely disappeared on the follow-up MRI two weeks later (E, F).

**Figure 2:**
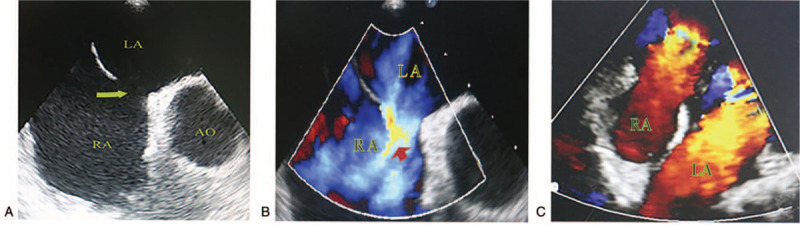
(A) Atrial septal defect (ASD) (arrow) was found on a transthoracic echocardiography. (B) Right-to-left shunts (RLS) were detected on color Doppler of transesophageal echocardiography (TEE) (arrow). (C) Postoperative TEE showed that the ASD was closed seamlessly, without blood flow. LA = left atrium, RA = right atrium, AO = aorta.

A 23-year-old man, with a medical history of pharyngitis for a week was admitted to our department with headache, drowsiness, occasional paraphasia, and paroxysmal dry cough. His body temperature was normal. Except for mild neck stiffness, no other neurological abnormalities were observed. A systolic murmur in 3 to 4 intercostals of the left sternum margin was heard when heart physical examination was performed. Brain magnetic resonance imaging (MRI) on admission showed an ovoid isolated lesion in the splenium of corpus callosum (SCC), which exhibited hyperintensity on diffusion-weighted imaging (DWI) and hypointensity on apparent diffusion coefficient (ADC) (Fig. 1 A and B). A discontinuous region (diameter: 14 mm) lying on the atrial septum was found by transthoracic echocardiography and was diagnosed as ASD (Fig. 2 A). Right-to-left shunts (RLS) were detected on color Doppler of transesophageal echocardiography (TEE) (Fig. 2B). Subsequently, microemboli were captured by transcranial Doppler ultrasound (TCD). His chest X-ray examination, magnetic resonance angiography, electroencephalogram, and electrocardiogram were unremarkable. Blood analysis, including blood cell count, electrolyte, and biochemical or coagulation indices were normal. Cerebrospinal fluid (CSF) showed a normal cell count, protein or glucose content with no elevated opening pressure. Virus-related antibodies (influenza virus A, B; rotavirus;measles;herpesvirus;adenovirus;or mumps virus), autoimmune encephalitis-related antibodies (NMDARAb, LGI1Ab, CASPR2Ab, GABABRAb, or AMPARAb), and bacteria culture were negative in serum and CSF. In the view of RLS and microemboli detected by TEE and TCD, respectively, oral aspirin 100 mg daily and lopidogrel sulfate 75 mg daily were administered to inhibit platelet aggregation (duration 14 days). In addition, oral nimodipine 40 mg daily to suppress vasoconstriction (duration 14 days) and oral 0.05 mg daily procarterol hydrochloride (duration 12 days) were administered to relieve his dry cough. On a review MRI performed on the seventh day of admission, the signal of the lesion in the SCC was weakened on DWI and ADC images (Fig. 1C and D), and completely disappeared on the follow-up MRI performed on the fourteenth day of adimission (Fig. 1E and F). All the symptoms resolved within two weeks. Subsequently, a repair surgery of ASD under thoracoscopy was successfully performed at our cardiac surgery department. Postoperative TEE showed that the ASD was closed seamlessly, without blood flow (Fig. 2C).

## 4 Discussion

RESLES, a recently identified clinico-radiological syndrome, has unknown etiology and its pathophysiological mechanism is poorly understood. To our knowledge, this is the first documented report of ASD as an underlying etiology for RESLES and discusses its pathogenesis.

In our patient, the initial brain MRI showed an ovoid lesion in the SCC with hyperintensity on DWI imaging and hypointensity on ADC imaging, which weakened on a review MRI a week later and completely disappeared on the follow-up MRI two weeks later. Mild neurological system disorders resolved after an effective treatment. Therefore, his clinical manifestations and imaging features met the diagnostic criteria of RESLES.^[1]^ Excluding the currently known etiologies such as infection, epilepsy, metabolic disorders, and so on, ASD seems to a plausible pathogenesis to explain his RESLES. Usually, RLS occurs in ASD patients in some special conditions, for example, when they have pulmonary hypertension or severe right heart impairment or have a cough or Valsalva maneuver.^[5]^ In these conditions, microemboli from the right atrium, normally filtrated in the lung, directly travel though the shunt, flow with blood to block some arterioles of the brain, finally lead to focal cerebral ischemia and cytotoxic edema.^[6]^ Moreover, some vasoactive substances such as serotonin, which are normally degraded by the lung, bypass the lung to trigger brain arterioles and cause vasoconstriction, which induce hypoperfusion of the local brain tissue prone to cytotoxic edema.^[7]^ Foong et al reported that the vasoconstriction of serotonin is more remarkable to arterioles than to main arteries such as the internal carotid and vertebral basilar artery.^[8]^ The artery supplying the SCC are tiny and tortuous^[9]^; hence, they are accessible to microemboli and sensitive to serotonin. With microemboli elimination and serotonin degradation, the blood supply of the SCC recovers quickly and cytotoxic edema disappears completely.^[6]^ Thus, brain MRI shows a reversible lesion in the SCC. In addition, because the damage is mild and transient, there is a good clinic prognosis. In our patient, ASD was found by transthoracic echocardiography on admission, RLS was detected by TEE when he coughed or had a Valsalva maneuver, and a microembolic signal was captured by TCD. The mechanisms, which usually explain the association of migraine and ASD,^[10]^ can similarly explain the relationship between RESLES and ASD of our patient.

The treatment of RESLES is controversial. Steroids and intravenous immunoglobulin have been recommended for patients with infectious etiology to suppress the inflammatory cytokines,^[1,11]^ but the effectiveness is still doubtful and unreasonable for patients with noninfectious etiology. Sometimes RESLES is regarded as a self-limited disease, without specific treatment.^[12]^ In our case, in view of the pathophysiological mechanism being closely associated with microemboli and serotonin, antiplatelet drugs and nimodipine were used to inhibit platelet aggregation and suppress vasoconstriction, respectively. It was effective for the patient as it led to his recovery.

In summary, to our best knowledge, this is the first case to report ASD associated with RESLES. Paradoxical microemboli and upregulation of serotonin may be the pathophysiological mechanism of RESLES. After treatment with antiplatelet drugs and nimodipine, the lesion of the SCC disappeared completely. The patient had a good prognosis. Therefore, ASD may be an underlying etiology for RESLES and need require an etiotropic treatment.

## Author contributions

**Conceptualization:** Jiangang Li

**Data curation:** Yingcong Chen

**Resources:** Jianxue Liu, Xingsheng Mai, Shaohua Jing **Writing – original draft:** Jiangang Li

**Writing – review & editing:** Jiangang Li, Yingcong Chen
